# High Prevalence of ESBL Genes in Commensal *Escherichia coli* of the Urinary Tract: Implications for Antibiotic Stewardship among Residents of Ghanaian Elderly Nursing Care Homes

**DOI:** 10.3390/genes15080985

**Published:** 2024-07-26

**Authors:** Emmanuel Armah, Lawrencia Osae-Nyarko, Bright Idun, Mawutor Kwame Ahiabu, Isaac Agyapong, Freda Boampong Kwarteng, Mercy Oppong, Naael Mohammed, Fleischer C. N. Kotey, Mike Yaw Osei-Atweneboana, Nicholas T. K. D. Dayie

**Affiliations:** 1 Biomedical and Public Health Research Unit, Water Research Institute, Council for Scientific and Industrial Research, Accra 00233, Ghana; emmanuelarmah44@yahoo.com (E.A.); lawkwarps@yahoo.com (L.O.-N.); bki27@yahoo.com (B.I.); mk_ahiabu@yahoo.com (M.K.A.); oheneagyapong@hotmail.com (I.A.); naanakwart21@gmail.com (F.B.K.); mercoppong50@gmail.com (M.O.); naeelsparrax@gmail.com (N.M.); mike.osei_atweneboana@csir.org.gh (M.Y.O.-A.); 2 Department of Medical Microbiology, University of Ghana Medical School, Korle Bu, P.O. Box KB 4236, Accra 00233, Ghana; fcnkotey@flerholiferesearch.com

**Keywords:** *Escherichia coli*, ESBL, uropathogen, antimicrobial resistance

## Abstract

The emergence and spread of extended-spectrum β-lactamase (ESBL)-producing *Escherichia coli* (*E. coli*) pose significant challenges to the treatment and control of urinary tract infections, particularly among vulnerable populations, such as the elderly living in nursing care homes. In this study, we investigated the occurrence of ESBL genes in commensal *E. coli* isolated from urine samples of 118 elderly individuals residing in Ghanaian nursing care homes. A total of 195 ESBL genes were detected among 41 *E. coli* isolated from the study participants. All the isolates harboured at least one ESBL gene, and the majority of them (70.1%) carried at least four ESBL genes. Among the ESBL genes detected, *CTXM825* was the predominant (14.1%). In antimicrobial susceptibility testing, 65.9% of the isolates showed resistance to cefepime, a fourth-generation cephalosporin, while 56.1% showed resistance to cefotaxime, a third-generation cephalosporin. Additionally, 46.3% of the isolates were multidrug-resistant, indicating resistance to antibiotics from multiple classes. In summary, we observed relatively high rates of resistance to antibiotics as well as alarming rates of ESBL genes in the isolated pathogens. These findings emphasise the urgent need for antimicrobial stewardship and infection control programmes to mitigate the spread of multidrug-resistant pathogens in nursing care homes.

## 1. Introduction

The urinary tract is a cardinal reservoir of antibiotic-resistant bacteria, which are often linked to urinary tract infections (UTIs) and related complications, such as sepsis, particularly among older adults [1]. In geriatric populations, *Escherichia coli* (*E. coli*) is the most commonly encountered uropathogen, and the emergence of extended-spectrum β-lactamases (ESBLs) among its strains has been a substantial hinderance to effective *E. coli* UTI management [1,2,3]. ESBL-producing *E. coli* strains are known to break down a broad range of antibiotics (including last-resort drugs like third-generation cephalosporins and carbapenems), conferring antimicrobial resistance (AMR) to carrier *E. coli* strains [1,2,3]. What is more intriguing is that *E. coli* AMR differs within and across various populations and demographics [4]. While much attention has been focused on uropathogenic *E. coli* strains, commensal *E. coli* strains also harbour ESBL genes that pose risks of disseminating antibiotic resistance across various bacterial genera [5,6,7,8,9,10]. To curb this challenge, it is important to understand the occurrence of ESBL genes in *E. coli* strains, particularly those isolated from vulnerable populations, such as older adults [11]. Notably, older adults patronise antibiotics the most and could serve as hubs for the development and spread of AMR *E. coli*; thus, focusing on them in connection with AMR surveillance will be essential in guiding antibiotic stewardship efforts and improving patient healthcare outcomes [11].

Despite the global recognition of ESBL-mediated *E. coli* AMR, limited data are available on the prevalence and implications of ESBL genes among elderly residents of nursing care homes in Ghana [12]. This is a significant vacuum in the healthcare data of Ghana, which may be largely due to a lack of adequate studies and surveillance of AMR among the elderly in nursing care homes. Of note, Ko et al. [13] emphasised the importance of recognising uropathogens and their resistance as a critical step towards improving the efficiency of treatment. Therefore, we investigated the extent of ESBL-mediated *E. coli* AMR among elderly residents of nursing care homes in Ghana. Specifically, we assessed the antibiotic susceptibility patterns of commensal *E. coli* strains isolated from the study participants, characterised the ESBL genes harboured by the pathogens, and determined the phylogenetic groupings of the isolates.

## 2. Materials and Methods

### 2.1. Study Sites and Sample Collection

A cross-sectional design was employed for this study, which was conducted between October 2022 and December 2022 and focused on 118 consenting elderly nursing care home residents in the Greater Accra Region of Ghana. Overall, there are twelve nursing care homes in Ghana (https://rentechdigital.com/smartscraper/business-report-details/ghana/nursing-homes [accessed on 10 October 2022]), and this study focused on five of them. These are located in two administrative districts of the Greater Accra Region of Ghana—Ga West and Tema Metropolitan—and host 200 residents in total. Early morning mid-stream urine samples were collected aseptically from the participants into sterile calibrated urine collection tubes. These were promptly transported to the medical microbiology laboratory of the Biomedical and Public Health Research Unit under the CSIR Water Research Institute in Ghana for bacterial and molecular analyses. Prior to sample collection, all recruited participants were anonymised with random code identifiers, and their demographic data (age and sex) were collected using a structured questionnaire. Individuals were excluded from the study if they did not fall within the age group of 55 to 100 years.

### 2.2. Bacterial Isolation and Identification

We cultured urine samples on cysteine–lactose–electrolyte-deficient (CLED) agar for primary bacteria isolation [14]. Colonies that appeared yellowish were presumed to be *E. coli* and were further cultured on 5% sheep blood and MacConkey agars. If more than one type of microorganism was isolated, they were recorded as “mixed cultures” and excluded from the study. The presence of such cultures is indicative of contamination from other sources and would lead to inaccurate identification and susceptibility testing. In cases where *E. coli* was found in mixed cultures, the sample collection was repeated under more stringent conditions to prevent contamination. Suspected *E. coli* isolates were subjected to biochemical tests for identity confirmation; these included the indole, methyl red, Voges-Paskeur, citrate, and triple sugar iron tests.

### 2.3. Antimicrobial Susceptibility Testing

The *E. coli* isolates were subjected to antimicrobial susceptibility testing (AST) based on the 2023 CLSI guidelines, and the results were interpreted using the same guidelines [15]. Briefly, a bacterial suspension was prepared, and its turbidity was adjusted to the 0.5 McFarland standard for comparison. The following antibiotics (Oxoid Ltd., Basingstoke, Hants, UK) were then tested against the bacteria: imipenem (IPM, 10 µg), ertapenem (ERT, 10 µg), aztreonam (AZM, 15 µg), cefepime (FEP, 30 µg), nitrofurantoin (F, 50 µg), cefuroxime (CXM, 10 µg), gentamicin (CN, 10 µg), amikacin (AK, 30 µg), ciprofloxacin (CIP, 5 µg), and levofloxacin (LEV, 10 µg). Isolates that were resistant to at least three categories of drugs were categorised as multidrug-resistant (MDR) [16].

### 2.4. DNA Extraction and Polymerase Chain Reaction (PCR)

#### 2.4.1. Screening for ESBL Genes

We used the QIAGEN extraction kit to extract the DNA of the pure colonies of *E. coli* based on the manufacturer’s protocol. Next, we subjected the extracted DNAs to primer-specific PCR to screen for 10 ESBL genes [17]. For each target gene, the PCR reaction mix was prepared to a volume of 10.0 µL per sample. This comprised 5 µL of 2× SYBR green master mix, 0.2 µL of each primer (forward and reverse), 2.6 µL of nuclease-free water, and 2 µL of DNA template. The PCR cycling conditions were set to an initial denaturation of 3 min at 94 °C, 30 cycles of denaturation for 45 s at 94 °C, 45 s at the various annealing temperatures, 1 min of extension at 68 °C, and a final extension step of 5 min at 68 °C. The primer sequences of the ESBL genes, amplicon sizes, and their respective annealing temperatures are listed in Table 1.

#### 2.4.2. Phylogenetic Grouping

**Figure 1:**
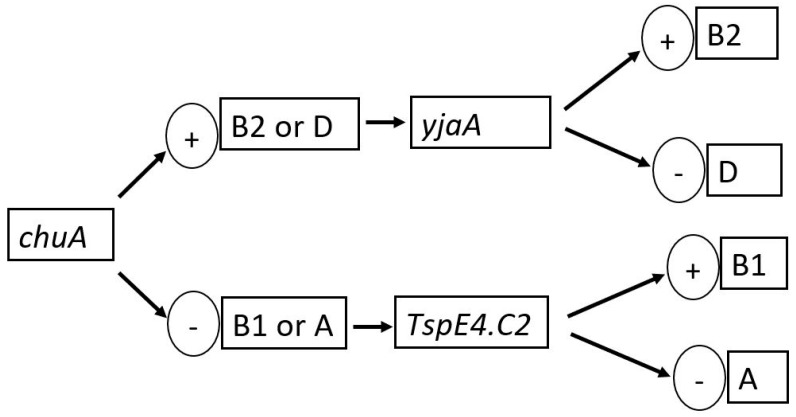
Dichotomous decision tree for phylogenetic group determination of the *E. coli* isolates.

We employed the triplex PCR method, previously described by Clermont et al. [18], in grouping *E. coli* isolates into phylogenetic clusters (A, B1, B2, and D) based on the presence or absence of specific genetic markers: *chuA*, *yjaA*, *TspE4.C2*, and *arpA*. Isolates belonging to Phylogenetic Group A typically carry the *chuA-*, *yjaA-*, and *TspE4.C2* markers and lack the *arpA* marker. Those classified into Phylogenetic Group B1 possess the *chuA-* and *TspE4.C2* markers, but lack the *yjaA* and *arpA* markers. Phylogenetic Group B2 isolates usually carry the *chuA* and *yjaA* markers, but lack the *TspE4.C2* and *arpA* markers, while Phylogenetic Group D isolates carry the *yjaA* and *arpA* markers but lack the *chuA* and *TspE4.C2* markers (Figure 1). The triplex PCR reaction mixture consisted of 5 µL of 2× SYBR green master mix, 0.2 µL of each primer, and 2 µL of DNA template. The PCR cycling conditions were set to an initial denaturation for 3 min at 94 °C, 30 cycles (denaturation for 45 s at 94 °C, annealing for 45 s at 59.2 °C, extension for 1 min at 68 °C), and a final extension step of 5 min at 68 °C. The marker-specific primer sequences and their amplicon sizes are listed in Table 2.

### 2.5. Statistical Analysis

The collected data, including demographic information, laboratory test results, and ESBL gene profiles, were stored in Microsoft Excel and imported into STATA 14 (Strata Corp., College Station, TX, USA) for analysis. The *p*-value for the distribution of ESBL gene carriage among *E. coli* isolates was calculated using the Chi-square test of independence. It was used to determine whether there is a significant association between the number of ESBL genes carried by the isolates and the observed frequencies.

### 2.6. Ethical Considerations

Ethical approval for this study was obtained from the Council for Scientific and Industrial Research—Institutional Review Board (CSIR-IRB), Ghana (Protocol ID: RPN 011/CSIR-IRB/2022). Participants provided oral and written consent prior to the commencement of the study. Permissions were also sought from the management and nurses of elderly care homes, as well as family members of these participants.

## 3. Results

### 3.1. Demographics of the Participants and the Prevalence of E. coli and ESBL Genes

The ages of the participants ranged from 55 to 99 years old. In total, 28.8% (*n* = 34) of the study participants were men, while 71.2% (*n* = 84) were females. A total of 58 (49.2%) of them were 70 years of age and below, while 60 of them (50.8%) were above 70 years of age.

**Figure 2:**
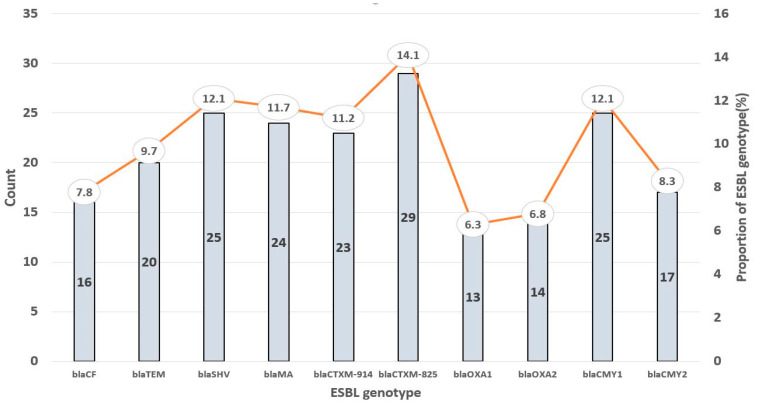
Prevalence of ESBL genotypes detected among the *E. coli* isolates.

Out of the 118 samples, 34.75% (*n* = 41) were positive for *E. coli*. These isolates yielded one hundred and ninety-five (195) ESBL genes, with *CTXM825* being the most detected/prevalent (14.1%, *n* = 29) and *OXA1* being the least occurring (6.3%, *n* = 13) (Figure 2). Also, a high prevalence of *CMY-1* (12.2%, *n* = 25), *SHV* (12.2%, *n* = 25), *MA* (11.7%, *n* = 24), and *CTXM-914* (11.2%, *n* = 23) genes was recorded. Furthermore, the ESBL genes *CF* (7.8%, *n* = 16), *TEM* (9.7%, *n* = 20), *OXA 2* (6.8%, *n* = 14), and *CMY-2* (8.3%, *n* = 17) appeared to have relatively low prevalence.

All 41 *E. coli* isolates were carriers of at least one ESBL gene: two (4.9%) were positive for only one ESBL gene, seven (17.1%) were positive for two ESBL genes, three (7.3%) harboured three ESBL genes, and twenty-nine (70.1%) were positive for at least four ESBL genes (Table 3). These differences were, however, not statistically significant (*p* > 0.05).

### 3.2. Antibiogram of the E. coli Isolates

**Figure 3:**
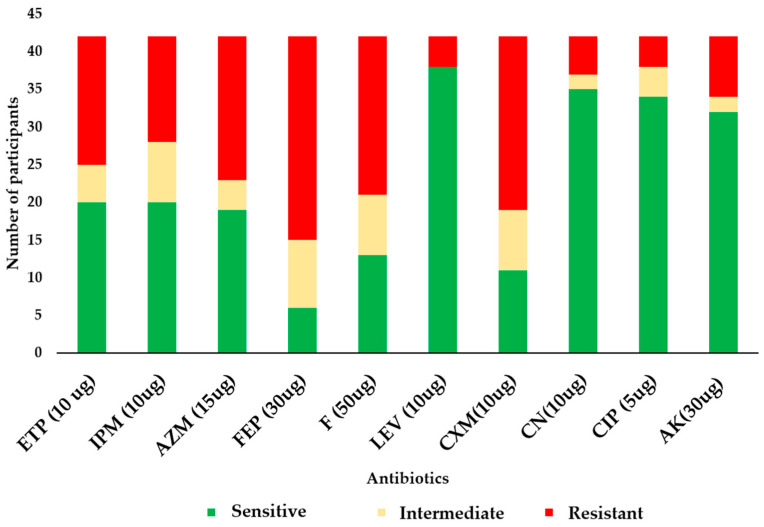
Antibiogram of the *E. coli* isolates. In the figure, ETP = ertapenem, IPM = imipenem, AZM = aztreonam, FEP = cefepime, F = nitrofurantoin, LEV = levofloxacin, CXM = cefuroxime, CN = gentamicin, CIP = ciprofloxacin, and AK = amikacin.

Resistance to cefepime was the highest in the study population, with 65.9% of the *E. coli* isolates showing resistance (Figure 3). The second-highest resistance was observed against cefotaxime, a third-generation cephalosporin, with 56.1% of *E. coli* isolates being resistant. This was followed by nitrofurantoin at a rate of 51.2%. Resistance rates against other important antibiotics decreased across aztreonam (51.2%, *n* = 21), ertapenem (46.3%, *n* = 19), imipenem (41.5%, *n* = 17), amikacin (34.1%, *n* = 14), gentamicin (19.5%, *n* = 8), ciprofloxacin (12.2%, *n* = 5), and levofloxacin (9.8%, *n* = 4). Additionally, 46.3% (*n* = 19) of the isolates were multidrug-resistant (MDR).

### 3.3. Phylogroups of the E. coli Isolates

The most prevalent phylogenetic group was Group A (62.3%, *n* = 28), followed by Phylogenetic Group B1 (26.8%, *n* = 11) and Phylogenetic Group D (4.9%, *n* = 2) (Table 4). No strains were assigned to Phylogenetic Group B2. Of the Phylogenetic Group A strains, 28.6% (*n* = 8) harboured the *yjaA* gene. All eleven Phylogenetic Group B1 strains (100%) harboured the *Tspe4.c2* gene, while 36.4% (*n* = 4) of them harboured the *yjaA* gene. Both Phylogenetic Group D strains harboured the *ChuA* and the *Tspe4.c2* genes (Table 4).

## 4. Discussion

This study investigated antibiotic resistance and the occurrence of ESBL genes among commensal *E. coli* strains isolated from the urinary tract of elderly nursing care homes in Ghana. Although some previous studies in Ghana have evaluated AMR and ESBL occurrence among *E. coli* occurring in urine, none of these have focused on elderly residents of nursing care homes [5,6,7,8,9,10,19]. To the best of our knowledge, this is the first such study among this population in Ghana, a major global AMR hotspot [3]. It helps fill a significant vacuum in the healthcare data of the country, as little to no data are available to support the development and implementation of appropriate guidelines for antibiotic usage in nursing homes in the country.

As observed, 34.75% of the 118 participants harboured *E. coli*, which concerningly carried 195 ESBL genes. The predominance of *CTXM825* among these genes is consistent with findings from studies involving clinical isolates of *E. coli* that highlighted widespread *CTX-M*-type ESBLs worldwide [20,21,22,23]. The concurrent high prevalence of the *CMY*-1, *SHV*, *MA*, and *CTXM*-914 genes highlights the extensive genetic heterogeneity of ESBL-producing *E. coli* strains and suggests the potential for multiple mechanisms of antibiotic resistance within the study population. Interestingly, *OXA1* was the least occurring ESBL gene, and *CF*, *TEM*, *OXA2*, and *CMY*-2 had relatively low prevalence. While these genes may be less prevalent in our study population, their presence and co-occurrence with other ESBL genes emphasise the complexity and potential for multiple mechanisms of antibiotic resistance within individual strains. These underscore the importance of comprehensive surveillance for ESBL genes to inform antibiotic stewardship efforts and guide empirical treatment strategies.

Not surprisingly, resistance to cefepime was the highest in the study population. Cefepime is commonly used to treat severe infections, and its elevated resistance raises concerns about the limited therapeutic options available for treating infections caused by ESBL-producing *E. coli* strains [20]. Of further concern, a high resistance rate was also observed against cefotaxime, a third-generation cephalosporin. Resistance to third-generation cephalosporins is a hallmark of ESBL-producing strains, emphasising the clinical significance of ESBL genes in the context of urinary tract infections [24]. Nitrofurantoin, which is commonly used for the treatment of urinary tract infections, recorded a similar high resistance rate, as did limited-use antibiotics like aztreonam (46.3%), ertapenem (41.5%), and imipenem (34.1%), further highlighting the extent to which treatment options against *E. coli* infections keep diminishing. These high resistance rates, especially the high MDR prevalence, highlight the need for careful consideration when selecting empirical treatment regimens.

Factors that may have contributed to the high prevalence of ESBL genes and MDR in the *E. coli* isolates in our study include pervasive overuse and misuse of antibiotics, inadequate infection control practices, age-related weak bladder muscles causing increased urine retention, and the widespread dissemination of resistant strains within healthcare facilities [25,26]. Addressing these factors requires a multifaceted approach, including antibiotic stewardship, enhanced surveillance, and the development of alternative treatment strategies, such as combination therapy and the use of non-antibiotic alternatives [27]. Continued monitoring and interventions are essential to mitigating the spread of antibiotic resistance and improving patient outcomes in this vulnerable population [28].

Phylogenetic group A, the most prevalent phylogenetic group among the *E. coli* isolates in this study, typically comprises commensal strains associated with the human gastrointestinal tract and is considered less virulent compared to other phylogenetic groups [18]. However, the detection of ESBL genes in group A strains suggests the potential for these strains to acquire and disseminate antibiotic resistance within healthcare settings [29]. Phylogenetic Group B1, the second-most frequent phylogenetic group, includes strains commonly associated with UTIs and may exhibit varying levels of virulence depending on the presence of specific virulence factors [30]. The detection of ESBL genes in Group B1 strains highlights the potential for these strains to cause UTIs and underscores the importance of surveillance and infection control measures to prevent the spread of antibiotic resistance [31]. Notably, no strains were assigned to Phylogenetic Group B2 in our study, and very few isolates belonged to Phylogenetic Group D. Phylogenetic group B2 is typically associated with extraintestinal pathogenic *E. coli* (ExPEC) strains, which are known for their ability to cause invasive infections such as bloodstream infections and UTIs [32]. While the absence of Group B2 strains may suggest a lower risk of severe infections in the study population, the presence of ESBL genes, as well as genes such as *yjaA*, *ChuA*, and *Tspe4.c2*, in other phylogenetic groups, calls for the need for continued monitoring and intervention to prevent the spread of antibiotic resistance [33].

This study had a couple of limitations. First, the number of sampling locations was five elderly homes in only two districts, and therefore, the findings may not necessarily reflect ESBL genes across all nursing homes in Accra and the country as a whole. Also, the sampling period for the study was relatively short, making it impossible to capture the variation in antibiotic resistance patterns across different seasons.

## 5. Conclusions

This study has provided valuable insights into the occurrence of ESBL genes and multidrug resistance among commensal *E. coli* strains recovered from elderly individuals living in nursing care homes in Ghana. The presence of ESBL genes in commensal *E. coli* strains underscores the potential for the dissemination of antibiotic resistance within care home environments, posing challenges for antibiotic surveillance and infection control measures. Resistance against several commonly used antibiotics was alarmingly high, emphasising the need for further studies to determine antimicrobial resistance mechanisms. Also, we recommend that further AMR studies and surveillance, prudent antibiotic prescribing practices, and antimicrobial stewardship programmes prioritise nursing care homes, as elderly residents are particularly prone to UTIs and can act as reservoirs for antimicrobial-resistant microbial strains.

## Figures and Tables

**Table 1:** Primer sequences of the ESBL genes, amplicon sizes, and their respective annealing temperatures.

Target Gene	5′–3′ Sequence	Size (bp)	Annealing Temperature (°C)
*bla*TEM	ATGAGTATTCAACAT TTC CG	840	55.1
	CCAATGCTTAATCAG TGA GG		
*bla*SHV	TTCGCCTGTGTATTATCTCCCTG	854	51.2
	TTAGCGTTGCCAGTGYTCG		
*bla*MA	ATGTGCAGYACCAGTAARGTKATGGC	593	51.2
	TGGGTRAARTARGTSACCAGAAYCAGCGG		
*bla*CTX-M 825	CGC TTT GCC ATG TGC AGC ACC	307	57.7
	GCT CAG TAC GAT CGA GCC		
*bla*CTX-M 914	GCT CAG TAC GAT CGA GCC	474	56.3
	GTA AGC TGA CGC AAC GTC TG		
*bla*CMY-1	GTGGTGGATGCCAGCATCC	915	56.1
	GGTCGAGCCGGTCTTGTTGAA		
*bla*CMY-2	GCACTTAGCCACCTATACGGCAG	758	52.5
	GCTTTTCAAGAATGCGCCAGG		
*bla*OXA-1	ATGAAAAACACAATACATATCAACTTCGC	820	51.2
	GTGTGTTTAGAATGGTGATCGCATT		
*bla*OXA-2	ACGATAGTTGTGGCAGACGAAC	602	56.1
	ATYCTGTTTGGCGTATCRATATTC		
CF	ATGATGAAAAAATCGTTATGC	1200	56.1
	TTGCAGCTTTTCAAGAATGCGC		

**Table 2:** The marker-specific primer sequences for the phylogenetic grouping and their respective amplicon sizes.

Target Gene	5′–3′ Sequence	Size (bp)
*TspE4.C2*	GAGTAATGTCGGGGCATTCA	840
	CGCGCCAACAAAGTATTACG	
*chuA*	GACGAACCAACGGTCAGGAT	854
	TGCCGCCAGTACCAAAGACA	
*yjaA*	TGAAGTGTCAGGAGACGCTG	593
	ATGGAGAATGCGTTCCTCAAC	

**Table 3:** Frequency of ESBL gene carriage among the *E. coli* isolates.

*E. coli* ESBL Gene Carriage	No. of Isolates (%)	*p* Value
Single gene	2 (4.9%)	1.0
Double genes	7 (17.1%)	
Triple genes	3 (7.3%)	
Multiple gene (≥4)	29 (70.1%)	

**Table 4:** Phylogenetic groups of the isolates.

Phylogenetic Groups	No. (%) of Isolates Positive for Amplification		
	*ChuA*	*vjaA*	*Tspe4.C2*
A (*n* = 28)	0	8 (28.6)	0
B1 (*n* = 11)	0	4 (36.4)	11 (100)
B2 (*n* = 0)	0	0	0
D (*n* = 2)	2 (100)	0	2 (100)

## Data Availability

The data underlying this study are available upon reasonable request from the corresponding author via ntkddayie@ug.edu.gh.
